# Extracellular vesicles package dsDNA to aggravate Crohn’s disease by activating the STING pathway

**DOI:** 10.1038/s41419-021-04101-z

**Published:** 2021-08-27

**Authors:** Fan Zhao, Tao Zheng, Wenbin Gong, Jie Wu, Haohao Xie, Weijie Li, Rui Zhang, Peizhao Liu, Juanhan Liu, Xiuwen Wu, Yun Zhao, Jianan Ren

**Affiliations:** 1 Research Institute of General Surgery, Jinling Hospital, Medical School of Nanjing University, Nanjing, People’s Republic of China; 2 Department of General Surgery, BenQ Medical Center, The Affiliated BenQ Hospital of Nanjing Medical University, Nanjing, People’s Republic of China

**Keywords:** Acute inflammation, Monocytes and macrophages, Signal transduction

## Abstract

Crohn’s disease (CD) is an intestinal immune-dysfunctional disease. Extracellular vesicles (EVs) are membrane-enclosed particles full of functional molecules, e.g., nuclear acids. Recently, EVs have been shown to participate in the development of CD by realizing intercellular communication among intestinal cells. However, the role of EVs carrying double-strand DNA (dsDNA) shed from sites of intestinal inflammation in CD has not been investigated. Here we isolated EVs from the plasma or colon lavage of murine colitis and CD patients. The level of exosomal dsDNA, including mtDNA and nDNA, significantly increased in murine colitis and active human CD, and was positively correlated with the disease activity. Moreover, the activation of the STING pathway was verified in CD. EVs from the plasma of active human CD triggered STING activation in macrophages in vitro. EVs from LPS-damaged colon epithelial cells were also shown to raise inflammation in macrophages via activating the STING pathway, but the effect disappeared after the removal of exosomal dsDNA. These findings were further confirmed in STING-deficient mice and macrophages. STING deficiency significantly ameliorated colitis. Besides, potential therapeutic effects of GW4869, an inhibitor of EVs release were assessed. The application of GW4869 successfully ameliorated murine colitis by inhibiting STING activation. In conclusion, exosomal dsDNA was found to promote intestinal inflammation via activating the STING pathway in macrophages and act as a potential mechanistic biomarker and therapeutic target of CD.

## Introduction

Crohn’s disease (CD) is a chronic transmural and relapsing inflammatory bowel disease, and is associated with an elevated risk of colorectal cancer [1–3]. Due to the incomplete understanding of its pathogenesis, CD remains incurable nowadays and usually accompanies patients for a lifetime, which results in a huge financial burden. In the past decades, literature has emerged that the imbalance of immune responses, e.g., the abnormal immune signals in macrophages, is strongly related to CD [1, 4]. Evidence suggests that the destruction of the immune microenvironment in CD mainly manifests as intestinal epithelial disruption, making intestinal epithelial cells an important source of damage-associated molecular patterns (DAMPs) [2, 5]. Macrophages can be activated by DAMPs and exhibit a pro-inflammatory subtype. Exploring the molecule mechanism behind the crosstalk of intestinal epithelial cells and macrophages can help promote understandings of CD.

In recent years, exosomes have become a hotspot in the field of biomedical research as an important cellular communication vector full of functional molecules. Exosomes are 30–150 nm extracellular vesicles (EVs) with lipid bilayer structures released by various cells in order to transfer small molecules to other cells [6–8]. The functional molecules encapsulated in EVs including nuclear acids, lipids, and proteins, largely decide its effect on the recipient cells. Extensive research has revealed that EVs are widely spread and can steadily exist in various body fluids including plasma, milk, and saliva [8, 9]. Easy access to EVs and its wide existence ensure EVs to be a potent biomarker and imply its regulatory roles in diseases including tumors, autoimmune and inflammatory diseases [10–17].

Previous studies have highlighted the role of EVs in CD to regulate intercellular signal transduction [18–20]. Studies have been performed on exosomal non-coding RNA such as lncRNA NEAT1 [21] and proteins like salivary exosomal PSMA7 [22]. Compared to exosomal RNA and proteins, little attention was paid to DNA within EVs, especially double-strand DNA (dsDNA), despite its pathogenic role in inflammatory and autoimmune diseases [23–27]. As an important DAMP in an anomalous intestinal microenvironment, dsDNA, including mitochondrial DNA (mtDNA) and nuclear genomic DNA (nDNA), has been shown to trigger innate immune responses by targeting cytosolic DNA sensors like STING to induce downstream signals [28, 29]. Although a recent study preliminary addressed the pathogenicity and upsurge of mtDNA in CD [30], the role of nDNA has not been discussed. Besides, there remains a paucity of research on the mechanistic details of dsDNA-mediated immunogenic responses. Given that nuclear acids can steadily exist in EVs, it is worth examining whether intestinal EVs transport pathogenic mtDNA and nDNA to trigger innate immune responses in CD.

The present research examined the emerging role of exosomal dsDNA in the context of CD. From our results, levels of dsDNA including nDNA and mtDNA within EVs in plasma or colon lavage were higher in active human CD and murine colitis, and both positively correlated with disease activity of CD. Besides, the concentration of dsDNA was significantly higher in EVs than in the remaining plasma after isolation of EVs, suggesting that the release of extracellular dsDNA mainly depended on EVs. Considering the extensive disruption of intestinal epithelial cells observed in murine colitis and active CD, we next determined damaged intestinal epithelial cells to be one of the important source cells of EVs. Mitochondrial damages inside intestinal epithelial cells and cell apoptosis in murine colitis accounted for the release of dsDNA. Correspondingly, a large number of EVs were shown to gather in the intestinal epithelium of active human CD. Therefore, EVs from damaged colon epithelial cells were employed in subsequent cell experiments to stimulate macrophages. By being internalized by macrophages, EVs transported dsDNA to trigger STING activation in macrophages, but failed after the removal of exosomal dsDNA. The result linked exosomal dsDNA with the STING pathway in macrophages and indicated the key role of dsDNA, instead of other bioactive molecules inside EVs. In line with the finding, the activation of the STING pathway was detected in the mucosal macrophages of active human CD. Meanwhile, inflammation was significantly alleviated in STING^−/−^ murine colitis and macrophages. The above findings illustrated the activation of the STING pathway by EVs in the development of CD. Lastly, the application of GW4869 to inhibit EVs release largely improved the disease prognosis of murine colitis by inhibiting the activation of the STING pathway. Taken together, our results unveiled that EVs transported pathogenic mtDNA and nDNA from damaged intestinal epithelial cells to macrophages in CD, activating the STING pathway to arouse inflammatory responses. The research first provided evidence about the effects of exosomal dsDNA and identified it to be a potent biomarker of disease activity for CD. EVs targeted therapy, like the application of GW4869 to inhibit EVs release, maybe a potential therapeutic trend for CD.

## Materials and methods

### Patients

The collection of plasma and tissue specimens during routine ileocolonoscopy from CD patients and trauma patients with no history of CD and no gastrointestinal symptoms were approved by the Ethics Committee of Jinling Hospital (Nanjing, China). All CD patients were stratified by Crohn’s disease activity index (CDAI), among which patients with CDAI greater than 150 were considered as active CD. Each patient provided written informed consent and patient data has been made anonymous. Characteristics of patients can be found in Supplementary Table 1.

### Mice

Wild type C57BL/6 and STING knockout (Tmem173^−/−^) mice were obtained from the Model Animals Research Center of Nanjing University, and housed in a temperature-controlled environment under a 12-h light/dark cycle. All mice within an experiment including WT and STING^−/−^ mice were cohoused for 4 weeks to minimize differences among microbiotas. Age (7–8 weeks) and sex-matched mice were used and randomly included in experimental groups. The animal study was designed and performed in strict accordance with the NIH Guide for the Care and Use of Laboratory Animals and with approval from the institutional animal ethical committee of Jinling Hospital.

### Murine experimental models

The experimental model of acute colitis was induced by 3% (w/v) dextran sodium sulfate (DSS) (M.W. 36,000–50,000, MPBiomedicals) in drinking water for 7 consecutive days, as described previously [31]. To inhibit the release of EVs during the modeling period, mice were injected 2.5 mg/kg GW4869 (M4974, AbMole BioScience) intraperitoneally on days 1, 3, and 5. Disease activity index (DAI) score was calculated daily, based on weight loss, stool consistency and the degree of intestinal bleeding as previously described [31, 32]. Each parameter was rated on a scale of 0–4 depending on severity, therefore the value of DAI would be within 0–12. The mice were sacrificed on day 8 for intestinal length measurement, histopathology, and measurement of cytokines. The histological score was calculated as previously described by a gastrointestinal pathologist expert blinded to the experiments [33]. Animal care and use were approved by Jinling Hospital Animal Care Committee.

### Histology, immunohistochemistry, TUNEL, and immunofluorescence assays

For histology, fresh intestinal mucosa specimens of human and colon tissues of mice were fixed in 4% paraformaldehyde, dehydrated in ethanol, embedded with paraffin, and dissected into 4-μm sections. Part of the slides were stained with hematoxylin and eosin (H&E) under standard conditions and sent to a gastrointestinal pathologist expert blinded to the experiments. The histological score was calculated as previously described [31].

For immunohistochemistry, the paraffin sections were deparaffinized in xylene, rehydrated in a concentration gradient of ethanol, undergone antigen retrieval, followed by incubating with the primary antibodies and secondary antibodies (Abcam), and then visualized with the DAB substrate kit (Abcam). Finally, the sections were counterstained with Mayer’s hematoxylin, rinsed with water, differentiated by alcohol, dehydrated, cleared, and mounted. The primary antibody of CD63 for staining of human intestinal mucosa used rabbit polyclonal antibody against CD63 (A5271, ABclonal).

TUNEL assays were performed using commercial kits (KGA703, Jiangsu KeyGEN BioTECH, Nanjing, China) in the colon tissues of murine experimental models according to the manufacturer’s instructions. The proportion of TUNEL-positive cells quantified the apoptotic index, as previously described [34]. In cell experiments, 30 μM Z-VAD-FMK (HY-16658B, MedChemExpress), a pan-caspase inhibitor, was administrated simultaneously with LPS to treat CT26 cells and TUNEL assays were performed in LPS-treated, LPS plus Z-VAD-treated and non-treated groups of CT26 cells respectively (KGA7071 for cell experiments, Jiangsu KeyGEN BioTECH, Nanjing, China).

For immunofluorescence staining, frozen sections were cut at 4 μm and mounted on slides, followed by incubating with 2% bovine serum albumin plus 1% newborn bovine serum in PBS for 60 min at room temperature to block the nonspecific background. Sections were incubated with CD68 antibody (GB14043, Servicebio) together with anti-Phospho-STING (phosphor Ser366, 85735, Cell Signaling Technology) or anti-Phospho-IRF3 (phosphor Ser396, 29047S, Cell Signaling Technology) at 4 °C overnight for human tissues. Nuclei were counterstained with DAPI (Jiangsu KeyGEN BioTECH, Nanjing, China). A confocal scanning microscope (FV1000, Olympus Corporation, Tokyo, Japan) was used for imaging analysis.

### Cytokine measurement

The protein levels of IFN-β (Nanjing Aoqing BioTECH, Nanjing, China), TNF-α (Cloud-Clone Corp, Wuhan, China), and IL-6 (Nanjing Aoqing BioTECH, Nanjing, China) in murine colon tissue homogenates were measured by commercially available ELISA kits according to the manufacturers’ instructions. mRNA levels of the cytokines in the cell culture pellet were detected by reverse transcription-quantitative polymerase chain reaction (RT-qPCR). All RNA samples obtained from cell medium were reverse-transcribed and quantified by Reverse Transcription Kit (R123-01, HiScript Q RT SuperMix for qPCR (+gDNA wiper), Vazyme International Corporation), and SYBR Green PCR Kit (Q711-02, ChamQTM Universal SYBR® qPCR Master Mix, Vazyme International Corporation) using the Agilent Bioanalyzer 2100 system (Agilent Technologies, CA, USA). Gene-specific primer sequences were listed in Supplementary Table 2. Results were normalized using GAPDH gene expression and shown as the relative expression value.

### EVs isolation

Fluid flushed from the 2-cm length of murine colons, namely colon lavage, was harvested using 0.5 ml PBS. Fresh blood samples were collected from retrobulbar venous plexus of mice and elbow vein of patients using anti-coagulant blood collection tubes, and centrifugated at 3000 × *g* for 10 min as soon as possible to obtain plasma. In terms of in vitro experiments, all cells were cultured in a commercial EVs-free cell culture medium (UR51101, Umibio, Shanghai, China) and the supernatants were collected after centrifugation at 1000 × *g* for 10 min. Before the isolation of EVs, processed colon lavage, plasma, and supernatants of cell culture medium were centrifuged at 3000 × *g* for 10 min, and subsequently centrifuged at 5000 × *g* for 10 min to remove the first stool, residual dead cells, cell debris, or other precipitate. The prepared supernatant fluid was next filtered using 0.8 µm pore and 0.22 µm pore sterile syringe filters (SLGP033RB, Millipore; SLAA025NB, Millipore) to remove bacteria or other living microorganisms. If necessary, the obtained fluid can be concentrated by centrifugation at 4000 × *g* using 100 kD ultrafiltration centrifuge tubes (UFC910024, Millipore). According to the guideline of Minimal Information for Studies of Extracellular Vesicles 2018 (MISEV2018) [6], the protocol of size-exclusion chromatography (SEC) and ultrafiltration was recommended as a method of high specificity for EVs isolation. Therefore a commercial kit (Echo9101A, Exosupur kit, Echobiotech) based on the principle of SEC was applied to isolate EVs according to the manufacturer’s instruction [35]. Briefly, the sample was loaded into the Sepharose-based CL-2B column which was prewashed with over 20 ml sterile PBS in advance. Then PBS was added into the column to eluate EVs after all samples were into the column. Each 500 μl of effluent represents one fraction and specific fractions enriched with EVs were collected according to the manufacturer’s instruction. The EVs enriched fractions were ultimately centrifugated at 4000 × *g* using 100kD ultrafiltration centrifuge tubes and stored at −80 °C for further studies.

### Isolation and quantification of exosomal mtDNA and nDNA

DNA was extracted from EVs using the QIAamp® DNA Blood Mini Kit (51104, Qiagen) and evaluated by Thermo Scientific™ NanoDrop™ 2000 Spectrophotometers. Quantitative polymerase chain reactions (qPCR) targeting Hist1h3F and mtCOI genes were performed to quantify nuclear genomic DNA (nDNA) and mitochondrial DNA (mtDNA) in murine models respectively. qPCR targeting H3 clustered histone 7 and mtCOI genes were performed to quantify nDNA and mtDNA of human samples respectively. Gene-specific primer sequences were listed in Supplementary Table 3. The efficiency of all reactions was >98%. All samples were analyzed in triplicate. The standard curve of the quantitative assay was produced through the serially diluted template cloned into a plasmid DNA.

### Transmission electron microscopy (TEM)

For assessment of mitochondrial morphology, colon tissues were washed with PBS and immediately transferred into a 3% EM grade glutaraldehyde solution before further processing. For ultrastructural comparative analysis, epithelial mitochondria were observed under TEM after Altmann staining.

For EVs identification under TEM, a total of 20 µl EVs enriched solution was placed on a copper mesh, incubated at room temperature for 5 min, and contrasted by 2% uranium dioxyacetate staining solution (Junrui BioTECH, Shanghai, China) for 1 min. After dried for 10 min at room temperature, the copper mesh was observed and photographed under a transmission electron microscope (H-7650C, Hitachi, Japan) at 80 kV. 30–150 nm cup-shape membrane-enclosed particles were identified as EVs.

### Nanoparticle-tracking analysis (NTA)

50 µl EVs enriched solution was first diluted by sterile PBS to a volume of 1 ml and then continuously diluted to determine ideal measurement concentrations. The size and distribution of particles were determined by ZetaView PMX 110 (Particle Metrix, Meerbusch, Germany) and analyzed by the corresponding software ZetaView 8.04.02 SP2. Each sample was repeated at least three times. NTA measurement was recorded and analyzed at 11 positions. The ZetaView system was calibrated using 110 nm polystyrene particles. The temperature was maintained at around 23 °C.

### Western blot

Colon tissues, cells, and EVs fractions were homogenized using the RIPA buffer (Jiangsu KeyGEN BioTECH, Nanjing, China), phosphatase inhibitor cocktail, and protease inhibitor cocktail (Jiangsu KeyGEN BioTECH, Nanjing, China). Pierce BCA Protein Assay Kit (Jiangsu KeyGEN BioTECH, Nanjing, China) was used to determine the protein concentration. Anti-CD63 (A5271, ABclonal), anti-Alix (12422-1-AP, Proteintech), anti-CD81 (66866-1-Ig, Proteintech), anti-Calnexin (ab22595, Abcam), anti-STING (19851-1-AP, Proteintech), anti-Phospho-STING (Ser366) (85735, Cell Signaling Technology), anti-Phospho-STING (Ser365) (72971, Cell Signaling Technology), anti-IRF3 (4302, Cell Signaling Technology), anti-Phospho-IRF3 (Ser396) (29047, Cell Signaling Technology), anti-NF-kB p65 (A19653, ABclonal), anti-Phospho-NF-kB p65 (Ser536) (3303, Cell Signaling Technology), anti-Beta-Actin (4970, Cell Signaling Technology), anti-GAPDH (AF1186, Beyotime Biotechnology) and secondary antibodies (KGP1201, Jiangsu KeyGEN BioTECH, Nanjing, China) were used for western blot.

### Bone marrow-derived macrophage preparation

Bone marrow cells were flushed from the femurs and tibias of C57BL/6 or STING-deficient mice and subsequently cultured with 10 ng/ml M-CSF (315-03, PeproTech, Cranbury, NJ, USA) and EVs-free cell culture medium (UR51101, Umibio, Shanghai, China) after centrifugation and excluding red blood cells. On the third day, 10 ng/ml M-CSF was added to the cell-cultured medium again. Four days later, the cells differentiated into bone marrow-derived macrophages (BMDMs) and were kept cultured in an EVs-free medium for further research.

### Flow cytometry

The leucocytes from the intestinal mucosa of colon tissues were isolated and stained with anti-CD11b (14-0112-82, eBioscience), anti-CD206 (25-2061-82, eBioscience), and anti-CD86 (14-0862-82, eBioscience). Data were acquired with a FACS Calibur (BD Biosciences) and analyzed using FlowJo software.

### EVs uptake assay and confocal microscopy

A murine colon adenocarcinoma cell line CT26 (Jianglin biotech, Shanghai) and BMDMs were employed. CT26 cells were cultured in 100-mm dishes (70–80% confluency) and treated with LPS (L2630, Sigma) for 24 h. EVs were next isolated from the supernatant of LPS-damaged CT26 cells and labeled with the membrane fluorescent dye PKH26 (UR52302, Umibo, Shanghai, China). EVs from untreated CT26 cells were also isolated and labeled. Subsequently, the isolation of EVs from the labeled EVs solution was performed again to exclude redundant dyestuff. Afterward, the labeled EVs were added to BMDMs for 15 h of incubation. After being washed by PBS thoroughly, cells were fixed in 4% paraformaldehyde for 10 min and stained with ActinGreen (KGMP001, Jiangsu KeyGEN BioTECH, Nanjing, China) and DAPI (Jiangsu KeyGEN BioTECH, Nanjing, China). Processed slides were observed using a confocal microscope (FV37, Olympus Corporation, Tokyo, Japan).

### Sonicated EVs and dsDNA digestion

In cell experiments, EVs fractions isolated from the culture medium of damaged CT26 cells were lysed by sonication using the ultrasonic cell disruptor (JY92-IIN, Guansen Biotechnology (Shanghai) Co., Ltd.). The process was repeated 6–8 times by 5 s pulse /5 s pause in an ice bath. To digest dsDNA inside EVs, dsDNase (EN0771, Thermo Scientific) was added to EVs fractions and incubated at 37°C for 30 min after sonication.

### Statistical analysis

Graph-pad prism 8 and R 3.53 were used for all statistical analyses. All experimental data were expressed in the form of mean ± SD. Differences among multiple groups were analyzed using one-way analysis of variance (ANOVA), followed by the least significant difference (LSD) post hoc test. By contrast, differences between the two groups were tested through the Student *t*-test. Meanwhile, Spearman r was used for correlation analysis. The survival rates in different groups were compared by the Kaplan–Meier test (log-rank). Quantitative data are presented as mean ± SD. A difference of *P* < 0.05 was considered statistically significant (*P < 0.05; **P < 0.01; ***P < 0.001).

## Results

### Abnormally elevated dsDNA levels in EVs were positively correlated with the disease activity of CD

**Fig. 1:**
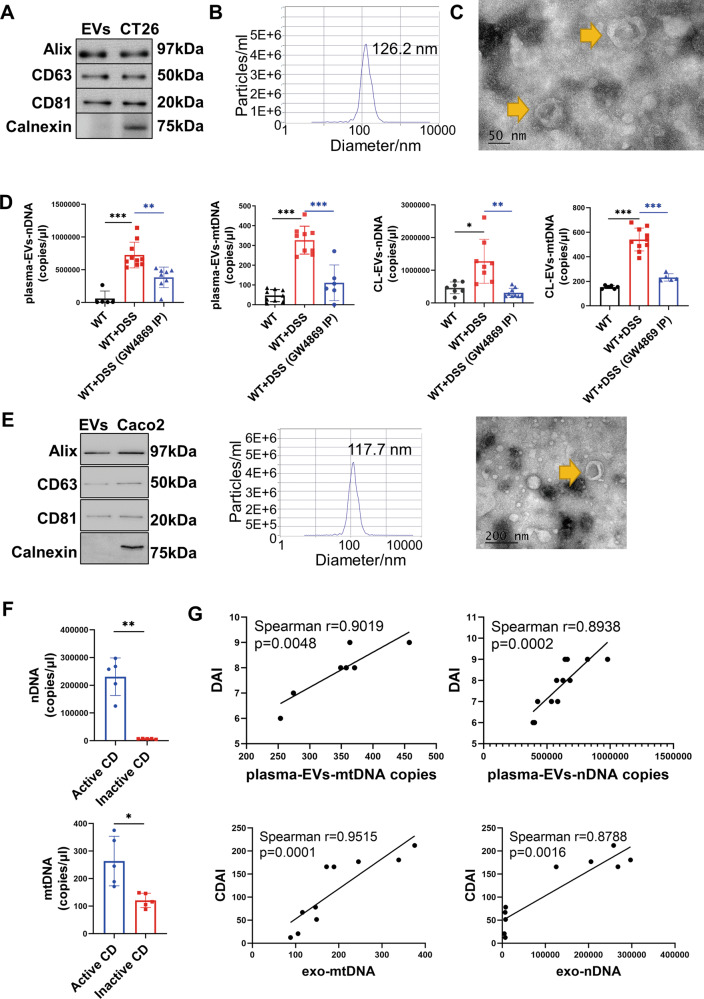
Abnormally elevated exosomal dsDNA was positively correlated with the disease activity in murine colitis and active human CD. **A**–**C** Classification of EVs in colon lavage of murine colitis. **A** Western blot of specific EVs markers: The simultaneous expression of three classic positive markers of EVs including Alix, CD63, and CD81, along with the absence of Calnexin, together identified EVs. The whole-cell lysate of CT26, a murine cell line, was used as a positive control. **B** Nanoparticle-tracking analysis of diluted EVs fractions was determined by ZetaView PMX 110 (Particle Metrix, Meerbusch, Germany). The distribution of EVs particle concentration (*y* axis) by size (*x* axis) was shown and 126.2-nm particles accounted for the highest proportion (98.5%). **C** Representative transmission electron microscope images of EVs fractions. The yellow arrow pointed to examples of EVs, which were cup-shape membrane-enclosed particles with diameters of 30–150 nm. **D** Absolute quantification of nDNA and mtDNA within EVs extracted from plasma and colon lavage of murine colitis. Hist1h3F gene and mtCOI gene were applied to represent nDNA and mtDNA respectively. Exosomal mtDNA and nDNA were significantly higher in the plasma and colon lavage of murine colitis. Murine colitis treated with GW4869 were also examined. *n* = 5–10/group. In the illustrations, healthy wild-type model, murine colitis, and murine colitis treated with GW4869 were abbreviated as WT, WT + DSS, and WT + DSS (GW4869 IP) respectively. Colon lavage was abbreviated as CL. **E** Classification of EVs isolated from plasma of active human CD, including western blot, nanoparticle-tracking analysis, and representative transmission electron microscope images. In the nanoparticle-tracking analysis, 117.7-nm particles accounted for the highest proportion (98.6%). In the representative transmission electron microscope image, the yellow arrow pointed to EVs. **F** Levels of exosomal nDNA and mtDNA from the plasma of CD patients were measured (*n* = 5/group). Both were significantly higher in patients with active CD. H3 clustered histone 7 gene and mtCOI gene were applied to represent nDNA and mtDNA respectively. **G** Correlations between the disease activity and levels of exosomal dsDNA in plasma in murine colitis and CD patients (Spearman’s rank correlation analysis). Disease activity index, Crohn’s Disease Activity Index, exosomal nDNA, and exosomal mtDNA were abbreviated as DAI, CDAI, exo-nDNA, and exo-mtDNA in the illustrations respectively. Data were displayed as mean values ± SD at least three independent experiments. *P < 0.05, **P < 0.01, ***P < 0.001.

To investigate whether EVs contain large amounts of dsDNA in active CD, samples from acute murine colitis and CD patients were both tested. The murine colitis model was established by adding 3% (w/v) DSS in drinking water for 7 consecutive days. EVs were extracted from both plasma and colon lavage using a recommended procedure based on size-exclusion chromatography (SEC) and ultrafiltration according to the MISEV2018 guideline [6]. In terms of EVs identification, western blot was firstly applied (Fig. 1A). Two tetraspanin proteins including CD63 and CD81, and endosomal sorting complex required for transport (ESCRT) accessory protein Alix, were chosen as positive markers of EVs. Meanwhile, calnexin was chosen as a negative marker of EVs according to the MIEV2018 guideline. Besides, cell lysates from the same species as EVs were set as a positive control. Apart from western blot, EVs were identified by transmission electron microscopy (TEM) and nanoparticle-tracking analysis (NTA) [6] (Fig. 1B, C). dsDNA was next extracted from EVs isolated from the colon lavage and plasma. Both Hist1h3f and mtCOI genes were examined by qPCR. Hist1h3f gene represents nuclear genomic DNA (nDNA) while mtCOI gene represents mitochondrial DNA (mtDNA). It turned out that the level of exosomal nDNA and mtDNA both increased significantly in EVs isolated from the colon lavage and plasma of DSS mice (Fig. 1D). To confirm the findings in murine colitis, EVs were isolated from the plasma of CD patients and identified (Fig. 1E). Exosomal mtDNA and nDNA both increased significantly in active CD patients (Fig. 1F).

Although a higher concentration of exosomal dsDNA in active CD has been clarified, it remains unclear whether dsDNA release mainly depends on EVs secretion. Therefore, dsDNA concentration was measured in the remaining plasma of murine colitis after isolation of EVs. The concentration of dsDNA outside EVs exhibited to be significantly lower than that in EVs (Supplementary Fig. 1A), which supports that dsDNA release mainly depends on EVs secretion.

Lastly, the level of exosomal dsDNA including nDNA and mtDNA in plasma was significantly correlated with disease activity index (DAI) in murine colitis and Crohn’s disease activity index (CDAI) in human CD (Fig. 1G), indicating the potential of exosomal dsDNA to reflect disease activity. Collectively, evidence suggested that both nDNA and mtDNA were sorted into EVs in CD, which may serve as a strong immune stimulant to initiate the development of intestinal colitis.

### EVs from damaged intestinal epithelial cells were internalized by macrophages to induce signaling

**Fig. 2:**
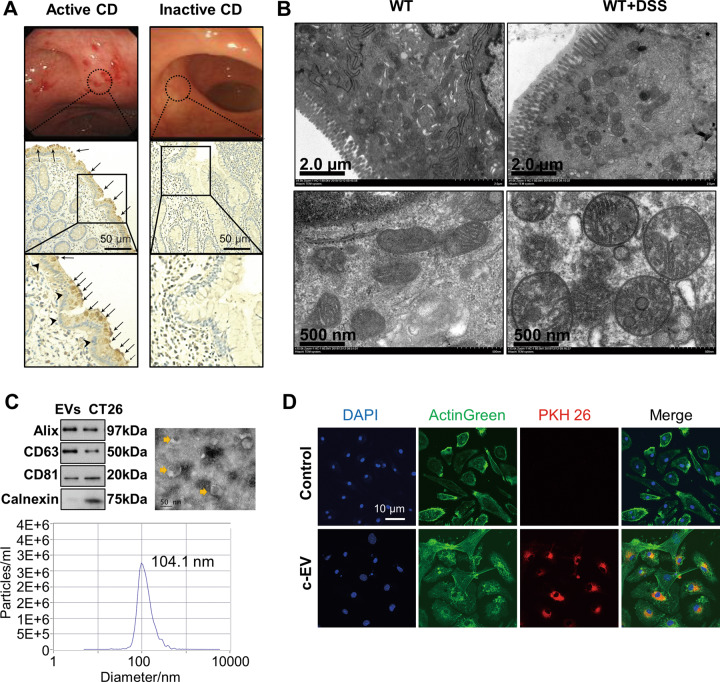
EVs from damaged intestinal epithelial cells were internalized by macrophages in vitro. **A** EVs presence in colons of human CD, exhibited by immunohistochemistry of CD63 (brown particles pointed by black arrows in the illustration), a transmembrane protein of EVs. The nuclei (blue) at the bottom of intestinal epithelial cells were pointed by black arrowheads. More EVs were stained in active CD and gathered in the intestinal epithelium. Colonoscopy images of corresponding patients were shown. **B** Transmission electron microscopy images of mitochondrial damages in colonic epithelia of murine colitis. Mitochondrial vesicular swelling and peripheral lucent zones were observed in the murine colitis model (abbreviated as WT + DSS in the illustration), compared to the wild-type healthy control. **C** Classification of EVs from LPS-damaged CT26 cells, including western blot, transmission electron microscopy, and nanoparticle-tracking analysis. The whole-cell lysate of CT26 was set as a positive control in the western blot experiment. In the nanoparticle-tracking analysis, 104.1-nm particles accounted for the highest proportion (98.5%). In the representative transmission electron microscope image, the yellow arrow pointed to EVs. **D** Labeled EVs were shown to be internalized by bone marrow-derived macrophages (BMDMs) after 15 h incubation. EVs from LPS-damaged CT26 cells were isolated and labeled with PKH26 (red). EVs from normal CT26 cells were used as control. BMDMs were treated with labeled EVs for 15 h and then observed under fluorescence confocal microscopy. F-actin (green) and nuclei (blue) of BMDMs were stained before the observation. EVs from damaged colon epithelial cells are abbreviated as c-EV in the illustration. All results were representative of at least three independent experiments.

EVs presence was examined in colon tissues obtained during ileocolonoscopy of CD patients by staining CD63, a transmembrane protein of EVs. Compared to inactive CD patients, a prominent increase of CD63 expression was detected in active CD, suggesting a massive release of EVs during active disease state (Fig. 2A). Quantification of EVs protein was also performed to represent the number of EVs. The expression of EVs markers including Alix, CD63, and CD81 was measured and turned out to be higher in the supernatants of LPS-treated CT26 cells than non-treated controls, which was in line with the higher level of cell apoptosis in the medium (Supplementary Fig. 2A, B). Consistently, 30 μM Z-VAD, a pan-caspase inhibitor, was administrated simultaneously with LPS to treat CT26 cells, and significantly decreased the level of cell apoptosis along with the expression of EVs markers in the system (Supplementary Fig. 2A, B). Besides, quantification of EVs proteins was also measured in the plasma of patients and turned out to be higher in active CD (Supplementary Fig. 2C). The above findings together illustrated that the secretion of EVs increased under inflammatory conditions.

The extensive damage to intestinal epithelial cells in CD indicates damaged intestinal epithelial cells to be an important source of EVs. The presence of EVs showed by staining of CD63 was mainly observed in the colonic epithelia of active CD (Fig. 2A), suggesting colonic epithelial cells to be a possible source of EVs. Besides, mitochondrial damages in colonic epithelia were observed in murine colitis models, including mitochondrial vesicular swelling, pyknosis, or peripheral lucent zones (Fig. 2B). The finding accounted for the leakage of mtDNA from intestinal epithelial cells. Along with our previous findings that the release of dsDNA mainly depended on EVs, mtDNA in the intestinal epithelial cells may be released via EVs. Therefore, although EVs could be secreted by various cells and intestinal epithelial cells did not have to be the only source cells, CT26 cells, a murine colon epithelial cell line, were employed as the donor cell for EVs in the subsequent cell experiments to determine the way EVs worked on macrophages.

Previous studies have shown that EVs can either be internalized by the recipient cell or adhere to the cell surface to induce signaling [13, 18]. To examine whether EVs were internalized by macrophages to arouse downstream effects, CT26 cells were treated with LPS to mirror the disruption of intestinal epithelial cells in CD. EVs were isolated from the supernatants of damaged CT26 cells and validated (Fig. 2C). After being labeled with PKH26, processed EVs were incubated with mature bone marrow-derived macrophages (BMDMs) for 15 h. Subsequently, labeled EVs were observed to be internalized by macrophages under the confocal microscopy, suggesting an uptake of EVs by macrophages (Fig. 2D). These findings provided evidence that EVs were internalized by target macrophages to elicit downstream effects.

### EVs activated STING pathway in macrophages in a dsDNA dependent manner to promote the development of CD

**Fig. 3:**
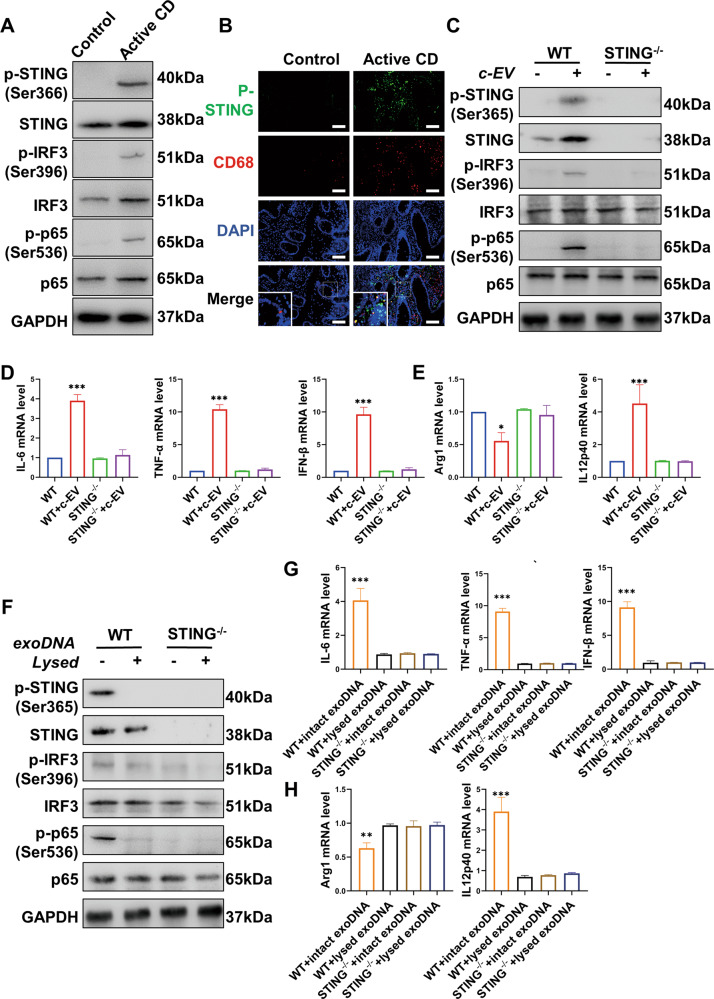
EVs activated the STING pathway and triggered macrophages to be pro-inflammatory in a dsDNA-dependent manner in CD. **A** Trauma patients with no history of CD and no gastrointestinal symptoms were used as controls. Expressions of p-STING, STING, p-IRF3, IRF3, p-p65, and p65 in colon tissues of active CD patients and controls were determined by western blot. The activation of the STING pathway was shown in active CD. **B** Immunofluorescence co-staining of CD68, a macrophage marker, and phosphorylated STING (p-STING) was performed in the colonic mucosa of active human CD. Nuclei were counterstained with DAPI. Trauma patients with no history of CD and no gastrointestinal symptoms were used as controls. Scale bar, 20 μm. **C**–**E** EVs from LPS-damaged CT26 cells were incubated with wild type (WT) or STING^−/−^ BMDMs. EVs were shown to trigger inflammation in macrophages by activating STING pathway. **C** Activation of STING pathway in BMDMs was determined by western blot. **D**, **E** mRNA levels of inflammatory cytokines, Arg1 and IL12p40 in BMDMs. The increased mRNA levels of IL-6, TNF-α, IFN-β, IL12p40 and decreased mRNA level of Arg1 showed that inflammation in macrophages was triggered by EVs from LPS-damaged CT26 cells. EVs from LPS-damaged CT26 cells are abbreviated as c-EV in the illustrations. **F**, **G** EVs from the equal number of damaged CT26 cells were assigned to two groups. One was lysed by sonication and digested with dsDNase so that exosomal dsDNA was digested thoroughly. The other group was directly treated with dsDNase to digest free dsDNA outside EVs, therefore exosomal dsDNA stayed intact. WT and STING^−/−^ BMDMs were treat with the two groups of EVs. Exosomal dsDNA was abbreviated as exoDNA in the illustrations. **F** Activation of STING pathway in BMDMs stimulated by the two groups of EVs. The EVs treated with dsDNase without sonication still activated the STING pathway in BMDMs while EVs treated with dsDNase plus sonication failed to. **G**, **H** mRNA levels of inflammatory cytokines, Arg1 and IL12p40 in BMDMs. EVs treated with dsDNase plus sonication failed to trigger inflammation in BMDMs. Data were displayed as mean values ± SD at least three independent experiments. *P < 0.05, **P < 0.01, ***P < 0.001.

The activation of the STING pathway in intestinal macrophages was investigated in active human CD. Trauma patients with no history of CD and no gastrointestinal symptoms were used as controls. The activation of the STING pathway in active CD patients was confirmed by western blot (Fig. 3A). By co-staining phosphorylated STING (p-STING) or phosphorylated IRF3 (p-IRF3) with CD68 (a marker of macrophages) in colon tissues respectively, higher expression of p-STING and p-IRF3 in mucosal macrophages was observed in patients with active CD (Fig. 3B and Supplementary Fig. 2D), confirming the activation of STING pathway.

In cell experiments, we next examined the activation of the STING pathway in macrophages by EVs. EVs from the plasma of active CD, inactive CD, and controls were isolated to stimulate BMDMs in vitro and STING activation was shown to be triggered by EVs from active CD (Supplementary Fig. 2E). EVs from damaged CT26 cells were also applied to BMDMs to examine downstream signals. As expected, apart from the activation of the STING pathway in macrophages (Fig. 3C) and higher expression of pro-inflammatory cytokines (Fig. 3D), the macrophage phenotype was also inclined to be pro-inflammatory (Fig. 3E). STING-deficient macrophages were also employed and EVs failed to trigger inflammation in this case (Fig. 3C–E). In conclusion, EVs from damaged intestinal epithelial cells activated the STING pathway in macrophages and subsequently aroused inflammatory responses in vitro.

**Fig. 4:**
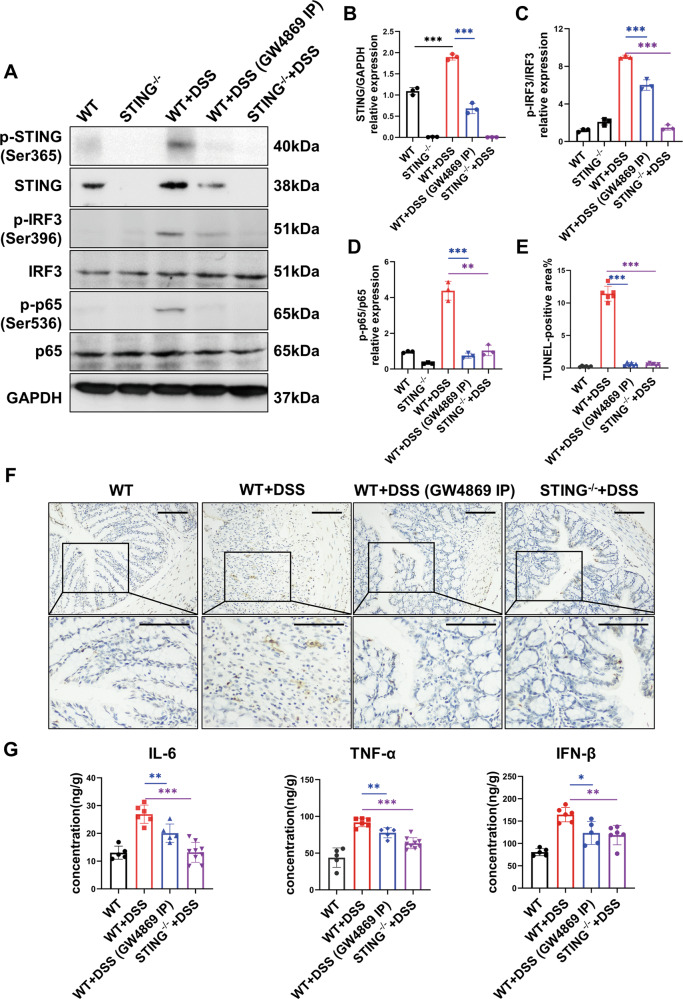
Blockade of EVs release by GW4869 inhibited intestinal inflammatory responses via inhibiting the activation of the STING pathway. **A**–**D** The expressions of p-STING, STING, p-IRF3, IRF3, p-p65, and p65 in colon tissues from Wild type (WT), STING^−/−^, WT murine colitis, WT murine colitis treated with GW4869, and STING^−/−^ murine colitis models were determined by western blot. WT murine colitis was abbreviated as WT + DSS in the illustrations. WT murine colitis treated with GW4869 was abbreviated as WT + DSS (GW4869 IP). STING^−/−^ murine colitis was abbreviated as WT + DSS. The activation of the STING pathway was observed in the WT murine colitis model and was inhibited in WT murine colitis treated with GW4869. **E**, **F** The proportion of TUNEL-positive cells was quantified to estimate intestinal apoptotic level in WT, WT murine colitis, WT murine colitis treated with GW4869, and STING^−/−^ murine colitis models. The level of apoptosis was higher in the WT murine colitis model and lower in WT murine colitis treated with GW4869, and STING^−/−^ murine colitis models. Representative images were shown. Scale bar = 50 µm. **G** The expression of inflammatory cytokines including IL-6, TNF-α, and IFN-β in the homogenates of murine colon tissues was measured by ELISA (*n* = 5–9/group). All results were representative of at least three independent experiments. Data were displayed as mean values ± SD. *P < 0.05, **P < 0.01, ***P < 0.001.

**Fig. 5:**
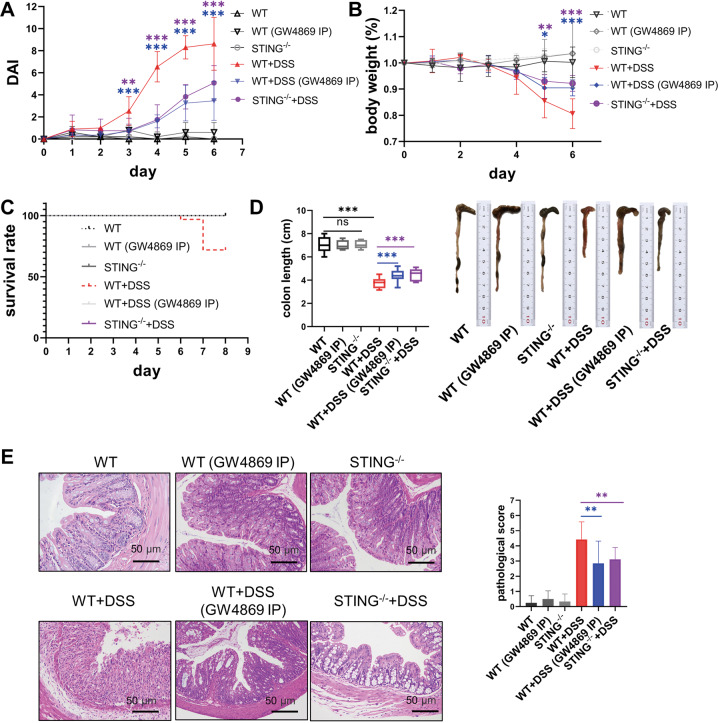
Either GW4869 administration to inhibit EVs release or STING deficiency improved the disease prognosis of murine colitis. **A**–**E** Comparison of disease prognosis among six experimental murine models (*n* = 11–20/group). WT (GW4869 IP): to examine possible side effects of GW4869, GW4869 was administrated in normal wild-type mice. WT + DSS (GW4869 IP): Murine colitis models treated with GW4869. STING^−^^/−^ + DSS: STING knockout (STING^−/−^) mice were employed and induced by DSS to establish colitis. Indicators of disease activity and severity were examined including **A** Disease activity index (DAI), **B** percent changes in body weight, **C** survival rate, **D** lengths and representative images of colons, **E** histological score of colons and representative colon H&E images. Data were displayed as mean values ± SD at least three independent experiments. *P < 0.05, **P < 0.01, ***P < 0.001.

To further verify the above findings in murine colitis, STING-deficient mice were employed to establish murine colitis. As expected, the level of intestinal apoptosis and pro-inflammatory cytokines both decreased compared to wild-type colitis mice (Fig. 4E–G). Furthermore, the prognosis of the model was significantly improved including disease activity and other prognostic indicators (Fig. 5A–E). Overall, these results confirmed the role of STING activation in exacerbating gut inflammation of CD.

Although there were various bioactive molecules in EVs, whether the STING pathway would be activated depends on the existence of exosomal dsDNA. To test this, EVs isolated from the supernatants of LPS-damaged CT26 cells were treated with dsDNase, sonication, or a combination of both before being applied to macrophages. The concentration of LPS, time of stimulation, the number of LPS-treated CT26 cells, and cell culture conditions such as culture dishes, the volume of culture medium, the CO_2_ concentration, and other conditions of the cell incubator, were, all the same, to ensure that equal number of EVs were obtained. As EVs are membrane-enclosed vesicles and can protect dsDNA inside EVs from the digestion of dsDNase, the application of dsDNase can only digest free dsDNA outside EVs. dsDNA within EVs still stayed intact. The sonication of EVs altered the permeability of the membrane, enabling dsDNase to digest exosomal dsDNA. Therefore the combination of sonication and dsDNase digested the existing dsDNA in or out of EVs. BMDMs were incubated with the processed EVs for 15 h, respectively, followed by western blot and qPCR to examine the activation of the STING pathway, inflammatory cytokine levels, and macrophage phenotype. Either EVs treated with dsDNase without sonication, or sonication without dsDNase can activate the STING pathway in macrophages, similar to the non-treated group (Supplementary Fig. 3A, B). Compared to the dsDNase-treated group in which exosomal dsDNA remained intact, EVs treated by the combination of sonication and dsDNase failed to trigger the activation of the STING pathway (Fig. 3F). Correspondingly, a marked increase in mRNA levels of inflammatory cytokines including TNF-α, IFN-β, and IL-6 was solely observed in the dsDNase-treated group with intact exosomal dsDNA (Fig. 3G). Macrophages were polarized toward a pro-inflammatory M1-type, while the effects disappeared after exosomal dsDNA was digested (Fig. 3H). Collectively, the results confirmed the pro-inflammatory role of exosomal dsDNA in vitro which activated the STING pathway in target macrophages and regulated macrophage phenotype.

### Blockade of EVs by GW4869 alleviated inflammation in murine colitis by inhibiting the activation of the STING pathway

To investigate the effect of EVs targeted therapy, GW4869 was applied in murine colitis to inhibit EVs release. Mice were intraperitoneally injected with 2.5 mg/kg GW4869 on days 1, 3, and day 5 during the 7 consecutive days of 3% DSS inducement (Supplementary Fig. 1B). EVs were isolated from plasma and colon lavage for analysis. Exosomal mtDNA and nDNA were quantified by qPCR, and both exhibited a significant shrinkage after inhibiting EVs release (Fig. 1D). Correspondingly, decreased expression of EVs positive markers suggested that EVs release was successfully inhibited by GW4869 (Supplementary Fig. 1C).

Given that a lower concentration of exosomal dsDNA was detected in murine colitis treated with GW4869 (Fig. 1D), we suspected that GW4869 inhibited the activation of the STING pathway to alleviate colitis. As expected, the STING pathway was significantly activated in murine colitis but largely inhibited after GW4869 treatment (Fig. 4A–D). Consistently, the gut inflammatory status was significantly decreased. TUNEL+ staining of the colonic mucosal displayed a decreased level of intestinal apoptosis (Fig. 4E, F). Inflammatory cytokines including TNF-α, IFN-β, and IL-6 were measured, and witnessed a significant decrease (Fig. 4G). Besides, there was a shift in the intestinal macrophage polarization towards an anti-inflammatory subtype in the GW4869 treated colitis group (Supplementary Fig. 4C, D). Together the findings provided evidence that the application of GW4869 to inhibit EVs release decreased the concentration of exosomal dsDNA and inhibited the activation of STING pathway and inflammatory responses in murine colitis.

The application of GW4869 improved the disease prognosis of murine colitis. A variety of prognostic indicators were significantly improved, including a remarkable improvement in the survival rate, colon lengths, and weight loss, as well as a significant shrinkage of DAI (Fig. 5A–D). The results were confirmed by histopathological changes in colons (Fig. 5E). Inflammatory cell infiltrating and crypt distortion were less observed after GW4869 treatment. The epithelium was more complete, indicating the reduction of epithelial cell death and abscission. Besides, age-matched mice intraperitoneally injected with 2.5 mg/kg GW4869 without DSS inducement were set as a control to test the biological toxicity of GW4869. From our results, this control group exhibited no significant differences in disease activity, bodyweight loss, survival rate, the length of colons, and intestinal pathological scores compared to normal wild-type mice, suggesting the safety of GW4869 application (Fig. 5A–E). In summary, these findings confirmed the safety of GW4869 application, and illustrated the significant improvement of disease prognosis in murine colitis treated with GW4869.

## Discussion

Endogenous DAMPs leaked from damaged intestinal epithelium play a key role in the development of CD by activating immune cells in lamina propria [36]. A recent study has identified mtDNA as a new endogenous DAMP in CD [30]. However, the role of nDNA, which is often leaked along with mtDNA during host cellular stress or injury, has not been clarified. In addition, whether nDNA and mtDNA are released directly or packaged by extracellular vesicles remains to be clear. Herein, our study revealed that the concentration of exosomal dsDNA, including nDNA and mtDNA, was significantly higher than that outside EVs, indicating that the release of dsDNA mainly depends on EVs. Besides, exosomal nDNA and mtDNA were both positively correlated with the disease activity of CD, which further activated the STING pathway, triggered macrophages to be pro-inflammatory, and augmented intestinal inflammation in CD. Application of GW4869 to inhibit EVs release significantly alleviated murine colitis by decreasing the concentration of exosomal dsDNA and inhibiting STING activation.

EVs derived from various cells have been reported to take part in cell-to-cell communication and regulate inflammatory responses [10–12]. The cargos of EVs include many different types of nucleic acids, lipids, and proteins. Previous studies have highlighted the pathogenic role of EVs in CD to regulate intercellular signal transduction [18–20], including exosomal non-coding RNA [21, 37] such as lncRNA NEAT1 [21] and proteins such as salivary exosomal PSMA7 [22]. However, there remains a paucity of research on exosomal dsDNA-mediated immunogenic responses in CD. Exosomal DNA has recently been investigated and plays a harmful role in other diseases such as viral infection, chemotherapy-induced inflammation, and tumors [24, 27, 38–40]. Our data first showed elevated levels of exosomal dsDNA in active CD. The disruption of intestinal epithelial cells, mitochondrial damages inside them, and EVs presence detected in the colonic epithelia of active CD, suggested damaged colonic epithelial cells to be one of the source cells of EVs in CD. In cell experiments, EVs derived from damaged intestinal epithelial cells were shown to transport dsDNA and internalized by macrophages to trigger inflammatory responses in a dsDNA-dependent manner. Together we provided evidence about EVs-mediated transfer of dsDNA between intestinal epithelial cells and macrophages in CD.

In terms of downstream mediators of exosomal dsDNA, we initially decided to determine the role of STING, a cytosolic DNA sensor, and also an adaptor molecule for numerous intracellular DNA receptors including cyclic GMP-AMP synthase, IFI16, and DDX41 [41–43]. The activation of the STING pathway was examined in active human CD, murine colitis, and macrophages stimulated by EVs. Besides, the activation of the STING pathway was inhibited in murine colitis after inhibiting EVs release. STING-deficient mice and macrophages were further employed in murine colitis and cell experiments. As expected, inflammation was largely attenuated in STING^−/−^ models. Our results were concordant with two recent studies demonstrating the pro-inflammatory role of STING in CD [44, 45], but contrary to Canesso’s study [29]. A few possibilities may account for the ambiguity, such as differences in the microbiome due to facilities or feeding environment, DSS source, and mice background.

Our study provided more possibilities for future research. Further studies are expected to investigate exosomal dsDNA-mediated responses in various diseases other than Crohn’s disease. EVs targeted therapy may be a future therapeutic trend for CD. Besides, apart from dsDNA, further investigations are required to elaborate the role of any other additional effectors or bioactive molecules in EVs, because EVs exert the overall functional effects as an ensemble.

## Conclusions

**Fig. 6:**
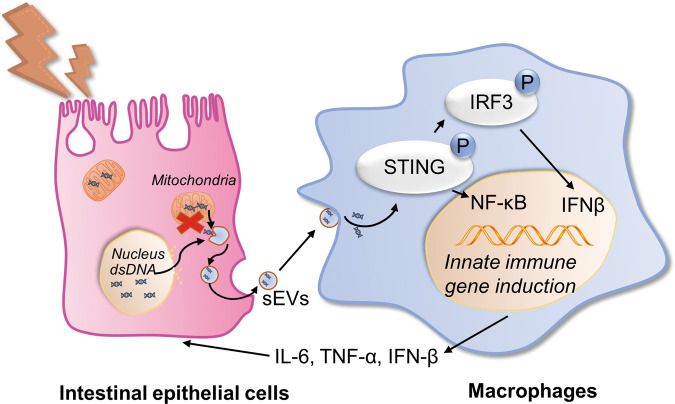
Model of exosomal dsDNA-mediated intestinal inflammation via activation of STING pathway in CD. sEVs full of dsDNA, which could be secreted by damaged intestinal epithelial cells, were taken up by macrophages to activate the STING pathway and trigger inflammation. Inflammatory cytokines released by macrophages, such as IL-6, TNF-α and IFN-β, further damaged intestinal epithelial cells.

The study sheds light on the critical role of exosomal dsDNA to activate the STING pathway in macrophages in CD, revealing a new mechanism of genetic exchange by EVs in the intestinal microenvironment (Fig. 6). Exosomal dsDNA in plasma is a new noninvasive indicator for the disease activity of CD. Administration of GW4869 to block EVs release can alleviate murine colitis by inhibiting the activation of the STING pathway. Our findings provide evidence that EVs targeted therapy might be a new therapeutic trend for CD in the future.

## Supplementary information

Supplementary materials

## Data Availability

The data underlying this article are available in the article and in its online supplementary material.
