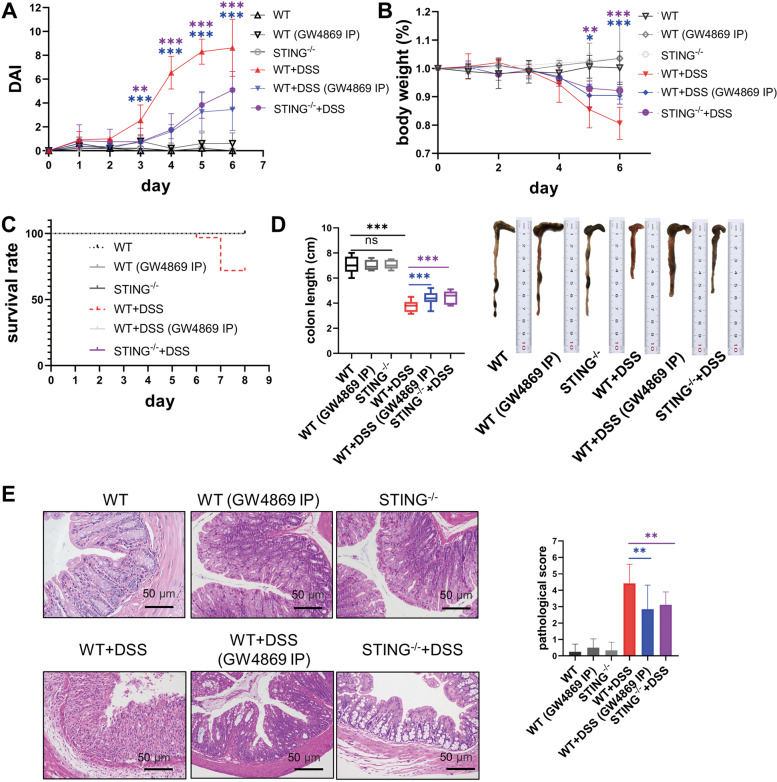# Correction: Extracellular vesicles package dsDNA to aggravate Crohn’s disease by activating the STING pathway

**DOI:** 10.1038/s41419-021-04340-0

**Published:** 2021-10-29

**Authors:** Fan Zhao, Tao Zheng, Wenbin Gong, Jie Wu, Haohao Xie, Weijie Li, Rui Zhang, Peizhao Liu, Juanhan Liu, Xiuwen Wu, Yun Zhao, Jianan Ren

**Affiliations:** 1 Research Institute of General Surgery, Jinling Hospital, Medical School of Nanjing University, Nanjing, People’s Republic of China; 2 Department of General Surgery, BenQ Medical Center, The Affiliated BenQ Hospital of Nanjing Medical University, Nanjing, People’s Republic of China

**Keywords:** Acute inflammation, Monocytes and macrophages, Signal transduction

Correction to: *Cell Death and Disease* 10.1038/s41419-021-04101-z, published online 27 August 2021

The original version of this article unfortunately contained a mistake. An image of the STING KO mice (without DSS) was mistakenly used for the representative colon H&E images for wild type mice (without DSS) during the compilation of Fig. 5E. The authors apologize for the error. The correct Fig. 5E can be found below.