# Supplementary material for: Extracellular vesicles package dsDNA to aggravate Crohn’s disease by activating the STING pathway
Source: Cell Death Dis. 2021 Aug 27;12(9):815. doi: 10.1038/s41419-021-04101-z (PMC8397775; doi:10.1038/s41419-021-04101-z)

**Supplementary information**

**Table 1** Demographic and main characteristics of the study population

|  | CD | non-CD controls |
| --- | --- | --- |
| Number, n | 10 | 9 |
| Median age, yr, (IQR) | 44.60 (34.25-52.00) | 46.78 (32.50-54.00) |
| Men, n (%) | 4 (40%) | 6 (67%) |
| Disease activity, n (%) |  |  |
| No | 10 | 9 |
| Clinical Remission (CDAI<150) | 5 (50%) |  |
| Clinically Active (CDAI≥150) | 5 (50%) |  |
| Disease location, n (%) |  |  |
| L1 | 1 (10%) |  |
| L2 | 7 (70%) |  |
| L3 | 2 (20%) |  |
| L4 | 0 |  |
| Disease behavior, n (%) |  |  |
| B1 | 0 |  |
| B2 | 5 (50%) |  |
| B3 | 3 (30%) |  |
| B2+B3 | 2 (20%) |  |
| Perianal lesions, n | 0 |  |

**Table 2** Primers for cell experiments

| Gene name (Species) | Primer sequences (5’ to 3’) | |
| --- | --- | --- |
| GAPDH (Mouse) | ACAACTTTGGCATTGTGGAA |  |
|  | GTCTTGTAGTAGGGACGTAG |  |
| Arg1 (Mouse) | AGTGTTGATGTCAGTGTGAGC |  |
|  | GAATGGAAGAGTCAGTGTGGT |  |
| IL12p40 (Mouse) | ACCCTGACCATCACTGTCAA |  |
|  | TGAGTGTAGACGACGAGGTG |  |
| TNF-α (Mouse) | GCATGATCCGCGACGTGGAA |  |
|  | AGATCCATGCCGTTGGCCAG |  |
| IFN-β (Mouse) | CTTGGGTGACATCCACGACTAC |  |
|  | GGCATAGCTGTTGTACTTCTTGTCTT |  |
| IL-6 (Mouse) | TTCACAAGTCCGGAGAGGAG |  |
|  | GAGCATTGGAAGTTGGGGTA |  |

**Table 3** Primers for quantification of mtDNA and nDNA

| Gene name (Species) | Primer sequences (5’ to 3’) | | |
| --- | --- | --- | --- |
| mtCOI (Human) | CGAGCTCGGTACCTCGCGAATACATCTAGAATGTTCGCCGACCGTTGACTATTCTCTACAAACCA | | |
|  | AGGCCTCTGCAGTCGACGGGCCCGGGATCCTCTAGATTTTAT  GTATACGGGTTCTTCGAATGTGT | |  |
| H3 Clustered Histone 7 (Human) | CGAGCTCGGTACCTCGCGAATACATCTAGAAGTTGTGACCATTGCTTGAAACCCATTCCTATGGC | |  |
|  | AGGCCTCTGCAGTCGACGGGCCCGGGATCCGGTGGCTCTGAAAAGAGCCTTTGGTTTAAGTTGG | |  |
| mtCOI (Mouse) | | CGAGCTCGGTACCTCGCGAATACATCTAGAATGTTCATTAATCGTTGATTATTCTCAACCAATCA | |
|  | | AGGCCTCTGCAGTCGACGGGCCCGGGATCCTTATTTTACTTTTACATAGGTTGGTTCCTCGAATG | |
| Hist1h3f (Mouse） | CGAGCTCGGTACCTCGCGAATACATCTAGAGTTGGGTGTGCCTGTTCGGTTTTATTTTGGTCCGG | |  |
|  | AGGCCTCTGCAGTCGACGGGCCCGGGATCCTGGAGGTGGCTCTTAAAAGAGCCGTTTTGGTTTAC | |  |

**Figure 1** Exosomal dsDNA was the main form of extracellular dsDNA. (A) Comparison of dsDNA concentration between EVs and the remaining plasma after isolation of EVs (n = 5/group). Exosomal dsDNA, was abbreviated as exoDNA in the illustration. dsDNA outside EVs was abbreviated as exofree-DNA. (B) The schematic diagram of murine colitis models treated with GW4869. Arrows indicated GW4869 administration (2.5 mg/kg, i.p.) on day 1, 3 and 5. (C) Expression of EVs positive markers was compared between EVs from murine colitis and colitis models treated with GW4869. Equal volume of plasma was used to isolate EVs. The decreased expression of EVs markers proved that EVs release was largely blocked by GW4869 administration. Data were displayed as mean values ± SD at least three independent experiments. *P < 0.05, **P < 0.01, ***P < 0.001.


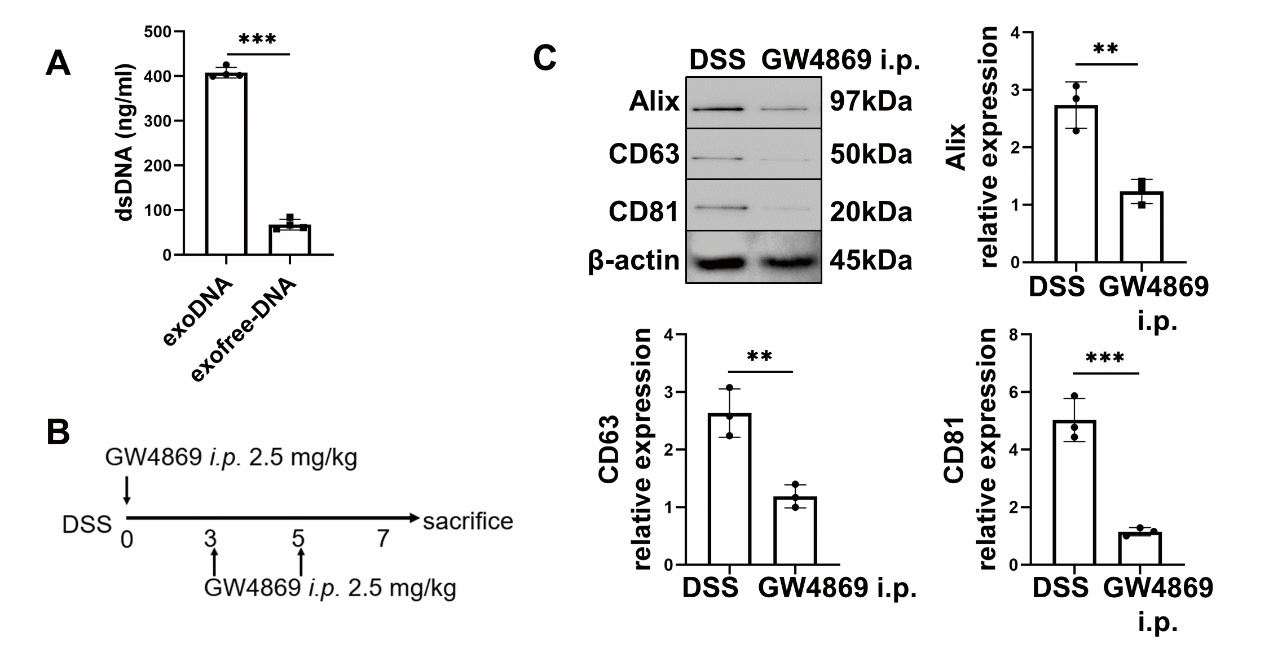


**Figure 2** EVs were proved to increase under inflammatory conditions, i.e. inflammatory cellular environment caused by LPS and CD patients during an active flare, which activated STING pathway in macrophages. (A) TUNEL assays detected the level of apoptosis in the groups of LPS, LPS plus Z-VAD, and non-treated CT26 cells. Representative images were shown. Scale bar, 50 μm. The red arrows indicated the apoptosis cells. The apoptosis level was significantly higher in LPS treated group and lower in the LPS plus Z-VAD group, as the application of Z-VAD inhibited the activity of caspase to inhibit cell apoptosis. 30 μM Z-VAD was applied in the experiment and 100 ng/ml LPS was applied to CT26 cells for 15 h. (B) EVs were isolated from the supernatants of non-treated, LPS-treated and LPS plus Z-VAD treated CT26 cells respectively. Equal amounts of protein (40-80 μg) of each EVs group were next separated on 10% SDS-PAGE to perform western blot. Quantification of three protein markers of EVs including Alix, CD63 and CD81 was performed to quantify EVs. The expression of EVs markers was higher in the LPS-treated group than non-treated group, revealing that the secretion of EVs increased in an inflammatory environment. Moreover, EVs protein quantification showed that the number of EVs decreased in the LPS+Z-VAD group, consistent with the decreased cell apoptosis and inflammatory levels. (C) Western blot was performed to detect the expression of Alix, CD63 and CD81 in EVs isolated from the plasma of active CD patients and controls. The result showed that the expression of EVs markers was higher in active CD who were during an acute inflammatory flare, indicating the increased secretion of EVs. (D) Immunofluorescence co-staining of CD68, a macrophage marker, and phosphorylated IRF3 (p-IRF3) was performed in the colonic mucosa of active human CD. Nuclei was counterstained with DAPI. Trauma patients with no history of CD and no gastrointestinal symptoms were used as control. As a downstream signal of STING pathway, the expression of p-IRF3 was shown in mucosal macrophages of active CD. Scale bar, 20 μm. (E) EVs were isolated from the plasma of controls, inactive CD and active CD to treat murine bone-marrow derived macrophages. Equal volume of plasma was used to isolate EVs. After 15 h incubation, the activation of STING pathway in macrophages was examined by western blot. Western blot analysis revealed that EVs from active CD significantly activated STING pathway in macrophages. All results were representative of at least three independent experiments. Data were displayed as mean values ± SD. *P < 0.05, **P < 0.01, ***P < 0.001.


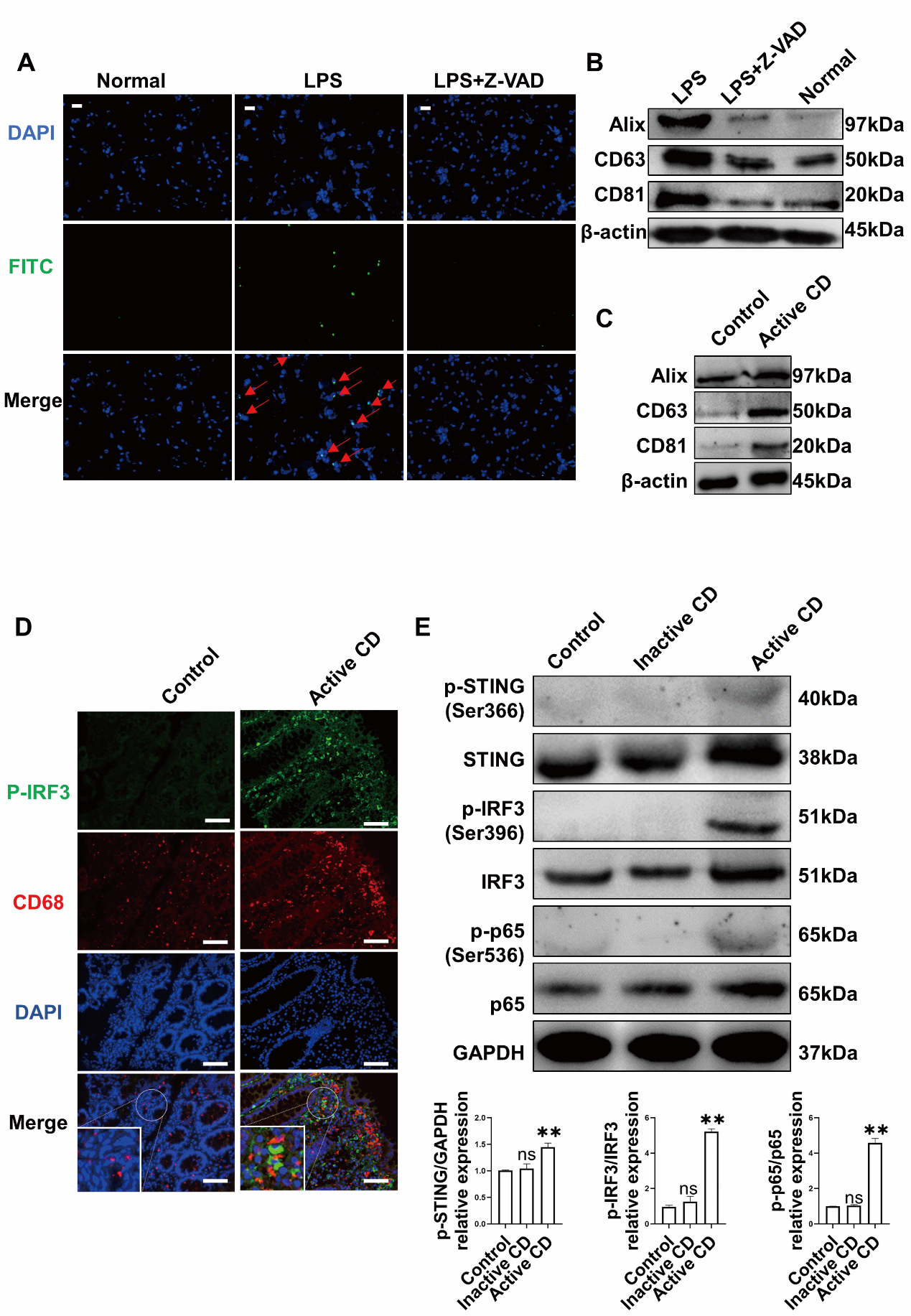


**Figure 3** The activation of STING pathway was detected in macrophages treated with dsDNase-digested, sonicated, sonicated plus dsDNase-digested, and non-treated EVs groups derived from LPS-damaged CT26 cells. (A) Activation of STING pathway in bone marrow-derived macrophages was determined by western blot after 15 h incubation with four groups of EVs derived from LPS-damaged CT26 cells. EVs derived from LPS-damaged CT26 cells, namely LPS-CT26-exo in the illustration, were treated with dsDNase, sonication or the combination of both before being applied to macrophages. The concentration of LPS, time of stimulation, the number of treated CT26 cells, and cell culture conditions such as culture dishes, the volume of culture medium, the CO_2_ concentration and other conditions of the cell incubator, were all the same to ensure that equal number of EVs were obtained in the four groups. (B) Densitometric quantification of the bands in the western blots showed that STING pathway was activated in macrophages treated with non-treated, sonicated, and dsDNase-digested groups of EVs, and was inhibited in macrophages treated with sonicated plus dsDNase-treated EVs. All results were representative of at least three independent experiments. Data were displayed as mean values ± SD. *P < 0.05, **P < 0.01, ***P < 0.001.


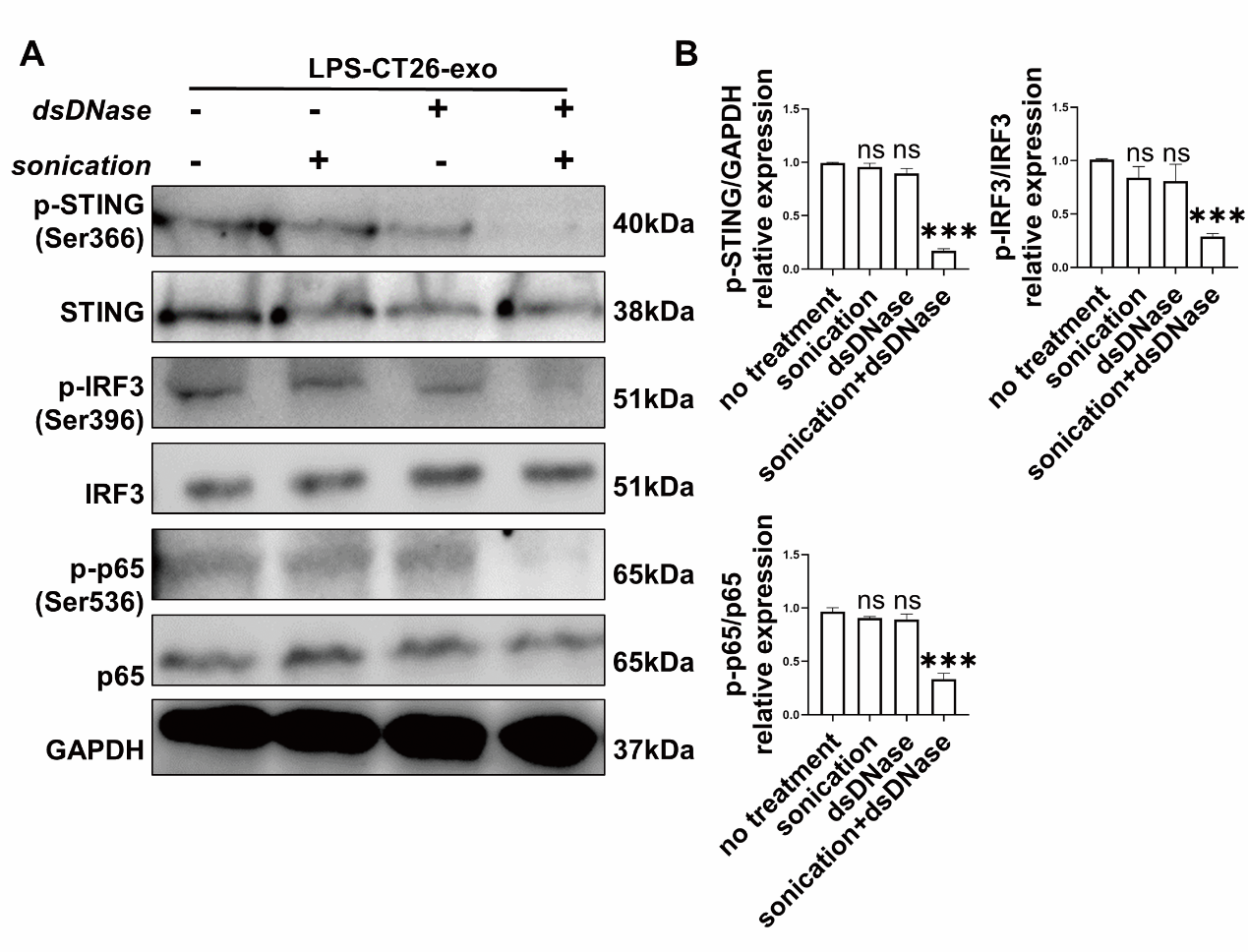


**Figure 4** Changes of small intestines and macrophage phenotype in murine colitis. (A)Transmission electron microscopy images of mitochondrial changes in small intestines of murine colitis. (B) Representative H&E images of small intestines in murine colitis. (C-D) Cellular fractions of macrophages in colonic mucosa of wild type and STING^−/−^ murine colitis, as well as wild type murine colitis treated with GW4869 were determined by flow cytometry (n=5-6/group). Representative dot plots were displayed. All results were representative of at least three independent experiments. Data were displayed as mean values ± SD. *P < 0.05, **P < 0.01, ***P < 0.001.


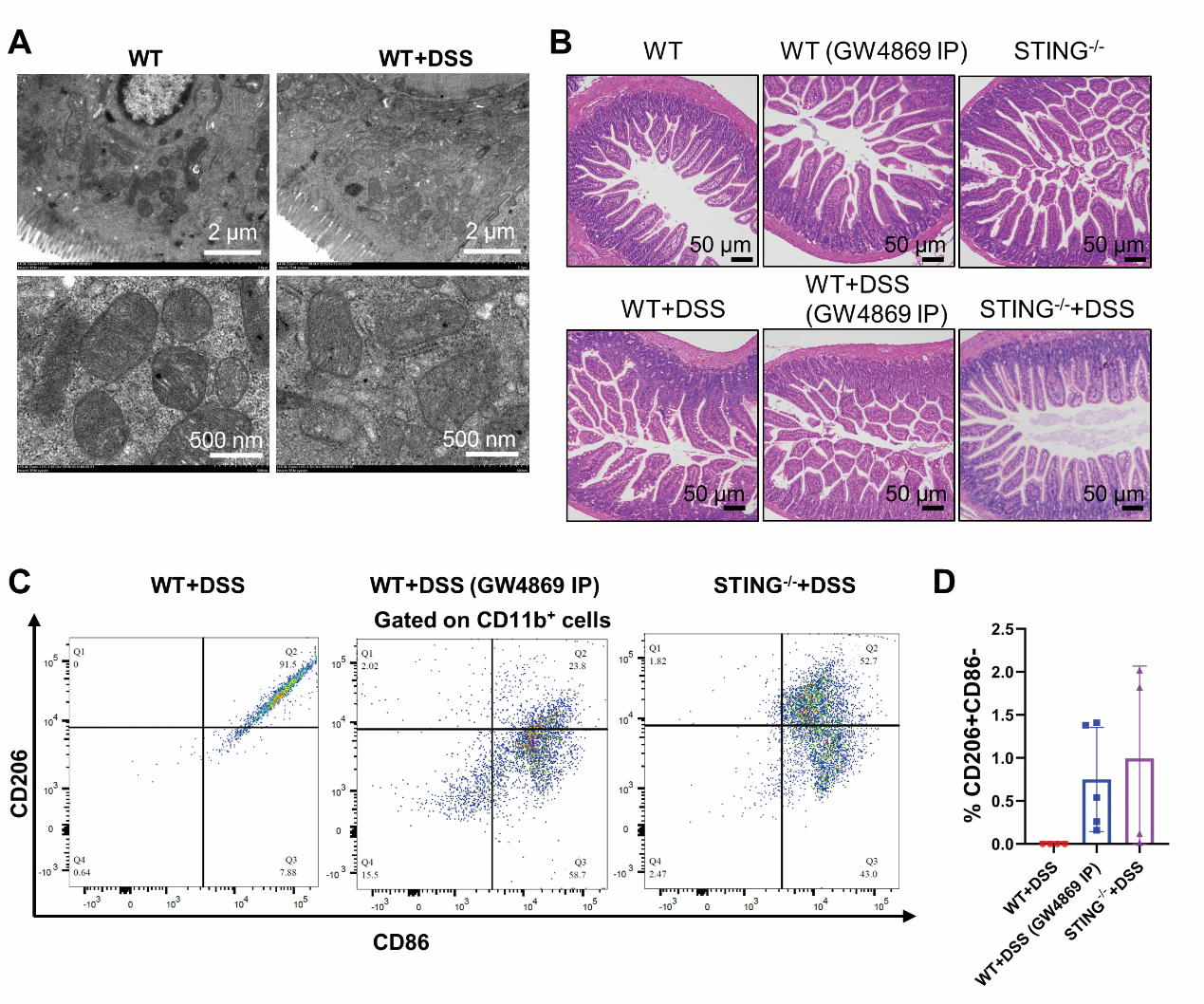

Supplement: Supplementary file 1 — Supplementary materials [file 41419_2021_4101_MOESM1_ESM.docx]
